# Supplementary material for: Thermoresponsive nanoemulsion-based gel synthesized through a low-energy process
Source: Nat Commun. 2019 Jun 21;10:2749. doi: 10.1038/s41467-019-10749-1 (PMC6588569; doi:10.1038/s41467-019-10749-1)
Supplement: Supplementary file 1 — Supplementary Information [file 41467_2019_10749_MOESM1_ESM.pdf]

Supporting Information

Thermoresponsive Nanoemulsion-Based Gel Synthesized Through a Low-Energy Process

Hashemnejad et al.

Supplementary Figures and Discussion

Optimizing the oil droplet size in the nanoemulsion. In order to minimize the oil (isopropyl myristate) droplet size in our nanoemulsion suspension, the HLB (hydrophilic lipophilic balance) value of the emulsifier (a mixture of Tween 80 and Span 80) was varied. The HLB value was estimated for a mixture of Tween 80 and Span 80 as HLB = 15x + 4.3(1-x), where x is the weight fraction of Tween 80, and 15 and 4.3 are the HLB values of Tween 80 and Span 80, respectively. Supplementary Figure 1a displays nanoemulsion (NE) hydrodynamic diameter and polydispersity index (PDI) of the formed nanoemulsion (without and with using polyethylene glycol 400, PEG 400, as a co-emulsifier) as a function of HLB of the mixed emulsifiers. Nanoemulsions were prepared using a low energy methodology, phase inversion composition (PIC), by adding dropwise continuous phase in to the magnetic stirring of the dispersed phase (oil, surfactants, and with or without PEG).[1] A nanoemulsion with an oil droplet diameter ~ 100 nm was formed at HLB ~ 13 (when PEG was not used). To further reduce the oil droplet size, polyethylene glycol (PEG) was utilized, which has previously been reported to act as a cosurfactant.[2] The molecular weight and concentration of the PEG molecule were systematically varied (Supplementary Figure 1). The added PEG, decreased the nanoemulsion droplet size. For example, using 5% wt. PEG 400 decreases the nanoemulsion droplet diameter from ~ 100 nm to ~ 50 nm (Supplementary Figure 1a). We also noticed that this effect occurs only if PEG was dissolved in the dispersed phase *during* the emulsification process. If PEG is instead added after nanoemulsion formation, it does not lead to a reduction in droplet size. This sensitivity to order of mixing of components is a key signature demonstrating the metastable nature of nanoemulsions, and distinguishes them from thermodynamically stable microemulsions, as discussed further in the next section.

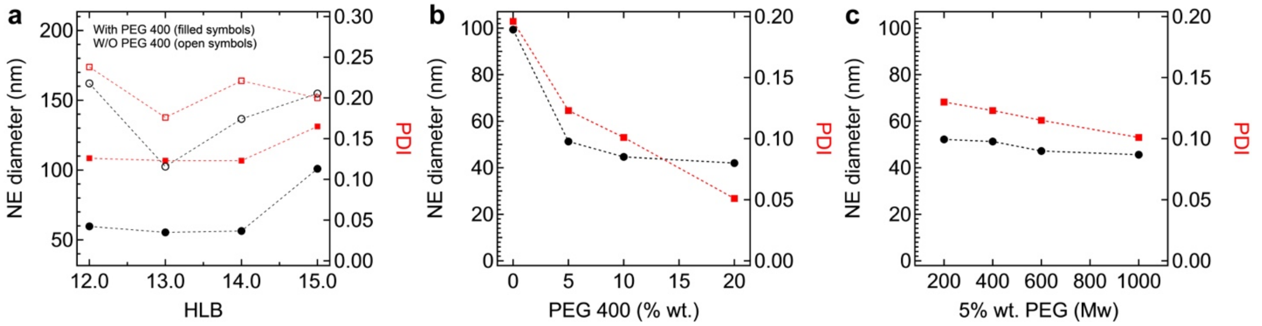

Supplementary Figure 1 Optimization of the oil droplet size in the nanoemulsion. Droplet diameter and polydispersity index as a function of **a)** HLB (hydrophilic lypophilic balance) for the oil-in-water nanoemulsion suspensions with (filled symbols) and without (open symbols) polyethylene oxide (PEG 400) added during formation, **b**) weight percentage of PEG 400 and **c)** molecular weight of the PEG molecule. Nanoemulsion suspensions were prepared through the low-energy method and contain 20% wt. (equivalent to volume fraction of ~ 0.24) oil (isopropyl myristate), 20% wt. surfactant (Tween 80 and Span 80) and 5% wt. cosurfactant PEG (unless otherwise specified), and remaining is DI water. The HLB value in b and c is 13.

Verification of the formed nanoemulsion and its stability. Nanoemulsions and microemulsions are both nano-sized droplets of an immiscible dispersed phase in a continuous phase using emulsifiers with amphiphilic properties. This similarity has led to much misinterpretation and confusion in the literature. Nanoemulsions are *kinetically* stable small droplets (typically <500nm). They are thermodynamically unstable. Conversely, microemulsions are in *thermodynamic equilibrium* and hence not a kinetically metastable state. Several recent review papers have been published to discuss the differences and

similarities between nanoemulsions and microemulsions, and different routes to discern these two systems from each other.[3] As aptly stated by Morales et al. in their 2003 article[4] comparing nanoemulsions to microemusions: “The similarities between these kinds of emulsions and microemulsions have been the origin of misinterpretations; some of the earlier reported microemulsions were not true thermodynamically but kinetically stable systems.”

Some preliminary tests can be performed in order to distinguish between a nanoemulsion versus a microemulsion, notably order of mixing of the components.[1] The microemulsions, which are thermodynamically stable systems, are not sensitive to their formation process, e.g. changing the order of mixing of the components. Whereas, our nanoemulsion (with hydrodynamic diameters of ~ 53 nm, for the canonical system) are only formed if emulsifiers (Tween 80, Span 80, and PEG 400 as a co-emulsifier) are first mixed with the oil (isopropyl myristate) prior to adding DI water. If the emulsifiers are first mixed in the continuous phase (water) before adding the oil a macro size emulsion or nanoemulsion with larger droplet size will be obtained. To verify this sensitivity to order of mixing, our canonical nanoemulsion were formed such that Tween80, Span 80, PEG400, and DI water were first mixed prior to dropwise adding isopropyl myristate into the magnet stirring of the continuous phase. Hydrodynamic size measurements indicated that oil droplet diameter increases to ~ 320 nm (with polydispersity index of 0.35). In addition, the formed nanoemulsion is unstable and after ~ 24 hours, an appearance of two distinct phases was observed. This sensitivity to order of mixing is a common feature when making nanoemulsions through a phase inversion point (PIT) method,[1] one type of so-called low energy method to make nanoemulsions.

Furthermore, microemulsions are strongly affected and often disrupted by changes in thermodynamic variables such as composition (e.g. dilution). In order to demonstrate stability upon dilution, our canonical oil-in-water nanoemulsion (with oil fraction of 0.24 and without addition of Pluronic) was diluted ~ 200 times in DI water and hydrodynamic diameters were measured as a function of time. Over the course of 24 hours we found that the droplet size increases only ~ 10% (Supplementary Figure 2a). We next investigated the stability of the droplets to temperature. Our canonical nanoemulsion system was heated to 50°C for several hours, and droplet diameters were monitored. The results indicated that droplet size in nanoemulsion increases ~ 37% of its original size after exposure to 50°C for 24 hr (Supplementary Figure 2a). It is expected that the rate of droplet size increase will be larger at elevated temperatures as the dominate mode for nanoemulsion size change is via Ostwald ripening. These experiments confirm our nanoemulsion is kinetically stable to changes in thermodynamic conditions.

To investigate the shelf life stability of the formed thermoresponsive nanoemulsion (as an end product for topical application), hydrodynamic size of the oil droplet was monitored at room temperature over the course of several months. Supplementary Figure 2b displays the hydrodynamic droplet diameter and PDI as a function of time. As it is shown, our temperature gelling nanoemulsion remains stable for over a year. Practically, long-term stability of the nanodroplets to storage is an important criteria in application of these colloidal suspensions.

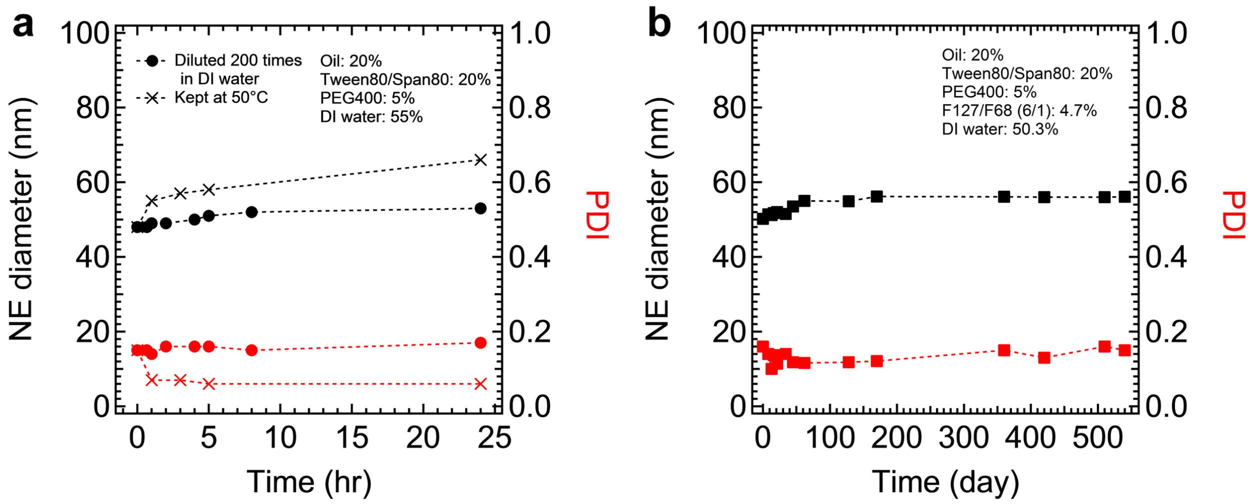

Supplementary Figure 2 Oil droplet size and polydispersity index, PDI, of the nanoemulsion (NE) as subject to dilution and elevated temperature as a function of time. Hydrodynamic diameters and PDI as a function of time for **a,** diluted NE with DI water (~ 200 times) and heated nanoemulsion suspension to 50°C (these nanoemulsions do not contain Pluronic). **b,** Long-term stability of thermogelling nanoemulsion (contains Pluronic) in the canonical formulation. The solution contains 20% wt. isopropyl myristate (equivalent to volume fraction of ~ 0.24), 20% surfactant (Tween 80 and Span 80; HLB = 13) and 5% wt. PEG 400 acting as a cosurfactant, the remaining of DI water. b contains 4.7% wt. Pluronic (mass ratio of F127/F68 is 6/1).

Control rheological measurements. The aim of the control rheology tests is to provide data that prove the key role of the oil droplets in the thermogelling behavior of our nanoemulsion formulation. Small amplitude oscillatory shear tests (Supplementary Figure 3a) and flow sweep tests (Supplementary Figure 3b) were performed to probe the sol-to-gel transition of the pure Pluronic mixture (F127/F68) utilized in this work. We found that the minimum concentration of Pluronic mixture to observe a sol-to-gel transition is ~ 13% wt. (and occurs at above 50 °C), and the shear elastic modulus (G') of the formed structure is only ~ 20 Pa at 60 °C. The same sol-to-gel transition was manifested in the flow sweep test for the pure Pluronic of 13% wt. at a temperature of approximately 50 °C. Therefore, these results confirmed that the lowest possible Pluronic concentration to observe a sol-to-gel transition is about 13% in the absence of added nanoemulsion. This concentration is much higher than the gelator concentration utilized in our canonical oil-in-water nanoemulsion and hence demonstrates that the oil droplets play an active and critical role in the gelation. These results also distinguish our work from prior studies in which the oil droplets are added to a continuous phase which would have gelled even in the absence of the added oil dispersion – i.e. lacking the synergistic affects we see in our system.

Rheological experiments were also performed on the continuous phase of the canonical nanoemulsion to provide additional information regarding the essential role of the oil droplets in the thermogelling behavior of the formulation. In these experiments we prepared an aqueous phase (no added oil dispersion) which was comparable to that of our canonical nanoemulsion system. Supplementary Figure 3c displays shear moduli (G' and G'', storage modulus and loss modulus) in a temperature ramp experiment (10 °C – 60 °C) of our canonical system (presented in Figure 2 in the main text) without presence of the oil. The concentration of the ingredients is similar to that shown in Figure 2 in the manuscript, except it contains no isopropyl myristate. In order to consider the local concentration of the Pluronic in our canonical system shown in Figure 2, the continuous phase was prepared such that it excludes the mass fraction of the oil. Supplementary Figure 3d displays the shear moduli as a function of

temperature of this continuous phase system. A second continuous phase was prepared by replacing the Span 80 with Tween 80 (with a similar equimolar amount), since Span 80 has a poor solubility in water. Supplementary Figure 3e displays G' and G'' as a function of temperature for the continuous phase of the canonical formulation where Span 80 was replaced by Tween 80. Overall in Supplementary Figure 3c, d, and e, we observe no sol-to-gel transition in the aqueous phases which mimic the conditions of the nanoemulsion continuous phase, further demonstrating that the presence of the oil droplets is key for the thermogelling behavior of our nanoemulsion formulation.

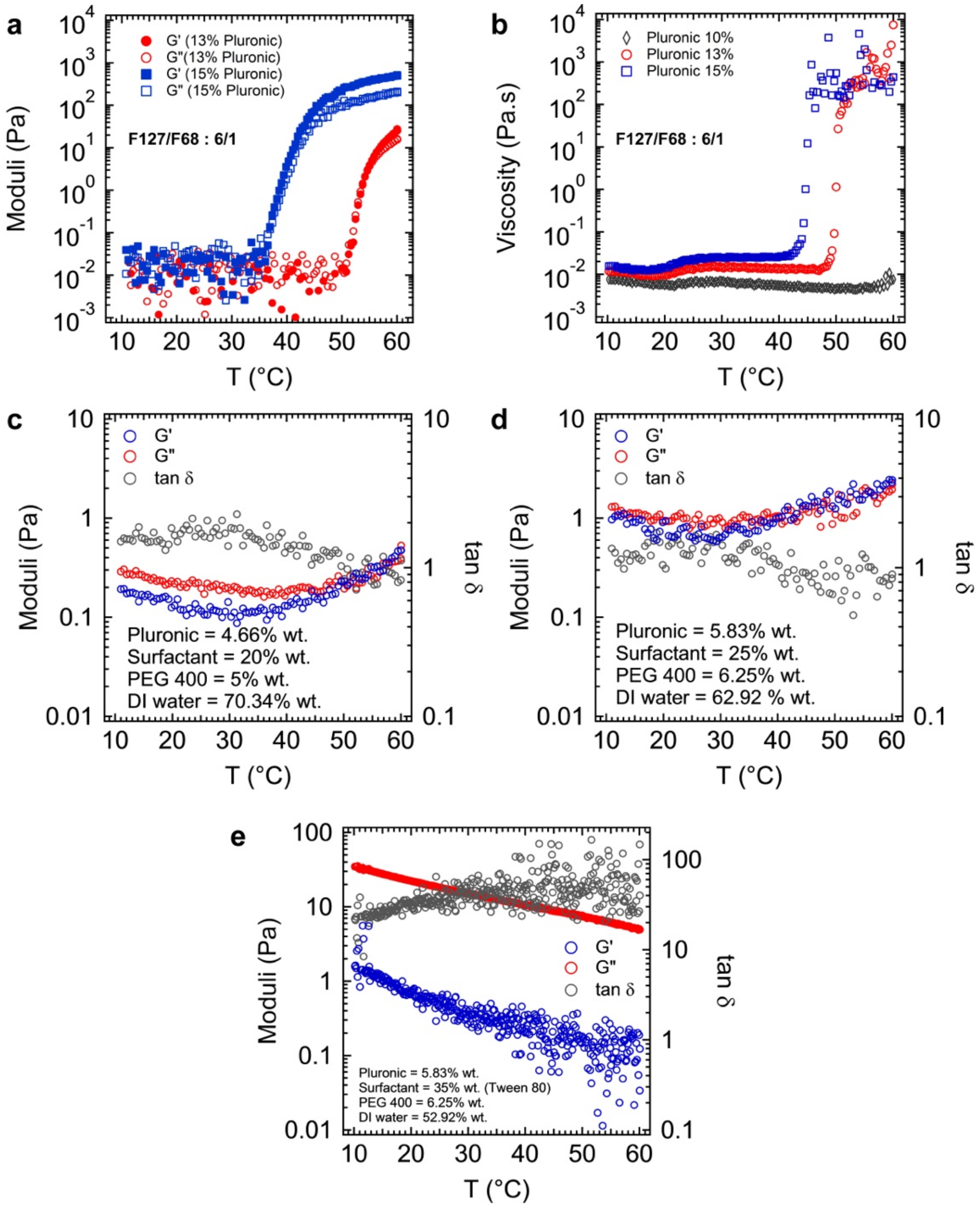

Supplementary Figure 3 Control shear rheology experiments without added nanoemulion. Shear moduli (**a**) and viscosity (**b**) as a function of temperature for different concentrations of aqueous Pluronic solutions (mass ratio of F127/F68 is 6/1). The viscoelastic moduli as a function of temperature for the continuous phase shown in Figure 2 in the main text: **c** is the exact concentration of the continuous phase noted in Figure 2, **d** is considering the local concentration by excluding the oil fraction, and **e** is the concentration of the ingredients where Span 80 was replaced

by Tween 80 (at similar molarity). The temperature ramp experiments (2 °C/min) were conducted at strain amplitude of 0.1% and frequency, ω, of 1 rad/s (lower frequency was selected to minimize the inertial effects). Flow sweep measurements were performed under applied stress of 0.1 Pa and temperature ramp rate of 2 °C/min.

Confocal microscopy. Confocal microscopy was used to image the microstructural evolution during the phase transition in our canonical thermogelling nanoemulsion. The imaging was carried out using a LSM 700 Zeiss Confocal Microscope equipped with an environmental chamber and diode lasers. An objective lens with magnification of 63X was used. In order to label the oil droplets, Nile red (a hydrophobic dye, at a concentration of ~ 0.05 mg in 1 mL isopropyl myristate) was dissolved in the oil phase during preparation of the thermogelling nanoemulsion. Supplementary Figure 4 illustrates the microstructure of the thermogelling nanoemulsion captured at liquid state (20 °C) and above the gel point (T_{gel} is ~ 35 °C). The obtained confocal images at above gel point is similar to that of liquid state. Unlike our previous thermogelling nanoemulsion with an arrested phase separation at the gel state (with characteristic length scales of microns),[5] the dispersed oil droplets of the canonical formulation remains homogeneous without formation of any visible micron size clusters. Due to the intrinsic resolution limits of the instruments (wavelength of the emitted laser is 550 nm), obtaining a higher resolution confocal image is not practical. The results from confocal experiments is consistent with the mechanism shown in Figure 1c.

We also do not believe that the gelation is due to depletion attraction of droplets. The Pluronic micelles are rather large (~20nm) and so from a colloidal point of view this system at high temperature is more akin to a bi-disperse suspension. Also, if the gelation was due to depletion, we would expect that doubling of the PEG concentration in Figure 5 to have a more dramatic effect on the plateau gel modulus.

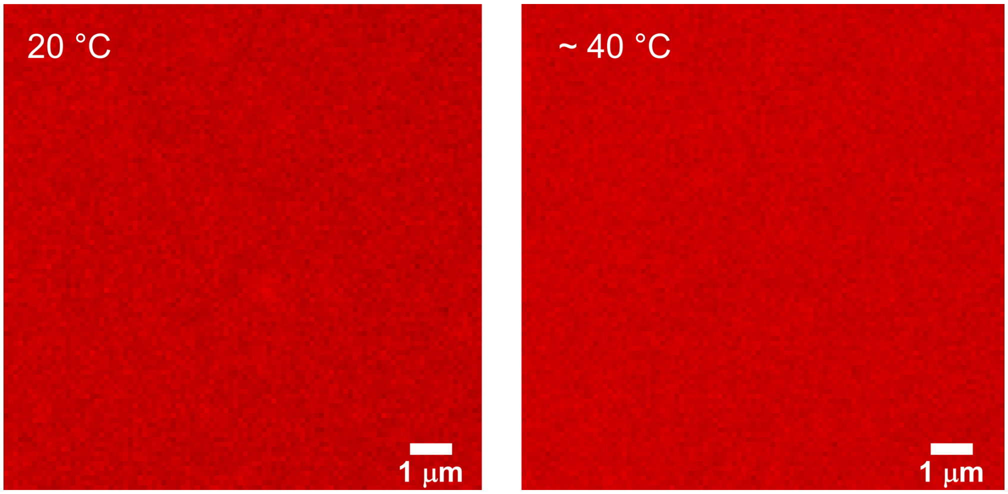

Supplementary Figure 4 Microstructure of the thermogelling nanoemulsion at ambient temperature an elevated temperature using confocal microscopy. Fluorescent regions belong to the oil droplets (volume fraction of oil is ~ 0.24). Composition of the sample is as follow: 20% wt. isopropyl myristate as the oil phase (contains 0.05 mg Nile red in 1 mL oil), 20% wt. surfactant as an emulsifier (a mixture of Tween 80 and Span 80 with HLB = 13), 5% wt. PEG 400, 4.7% wt. Pluronic (F127/F68: 6/1 g/g), and 50.3% DI water.

Small angle X-ray scattering. The scattering experiments were performed by staff at the Argonne National Lab.[6] The samples were loaded (using 22 gauge needle 6 in long metal) in quartz capillary with nominal outer diameter of 1.5 mm (Hampton Research Corp.), and wall thickness of 0.01 mm. The scattering profiles (combination of ultrasmall and small angle X-ray scattering, USAXS and SAXS) of a thermogelling nanoemulsion at liquid and gel states are displayed in Supplementary Figure 5. First, we expect that the scattering is dominated by the oil droplets. At intermediate q, both samples have a Porod slope of q^{-4} which is due to the spherical nanoemulsion droplets. The overlap of the two data sets in this Porod region confirms that the spherical droplets remain intact at elevated temperatures. At 25°C we observed a small peak at a scattering vector of ~ 0.015 $Å^{-1}$ which does not shift at 50°C. This peak is most likely due to the spatial correlation between neighboring droplets expected for nearly hard spheres at this volume fraction (0.24). Unlike our prior work[7] using bridging gelators with nanoemulsions, at low q we observed no evidence of droplet clusters forming in the gelled state (50°C) and the scattering profiles nearly overlap, though with the 50°C data slightly below the 25°C. The lack of significant structure seen in the scattering pattern in the gelled state is consistent with our confocal microscopy images (Supplementary Figure 4) and also consistent with our proposed gelation mechanism in which the droplets are intermixed with the Pluronic micelles. We also note that the scattering data supports our hypothesis that the gelation is not due to depletion-induced attraction of the nanoemulsion droplets.

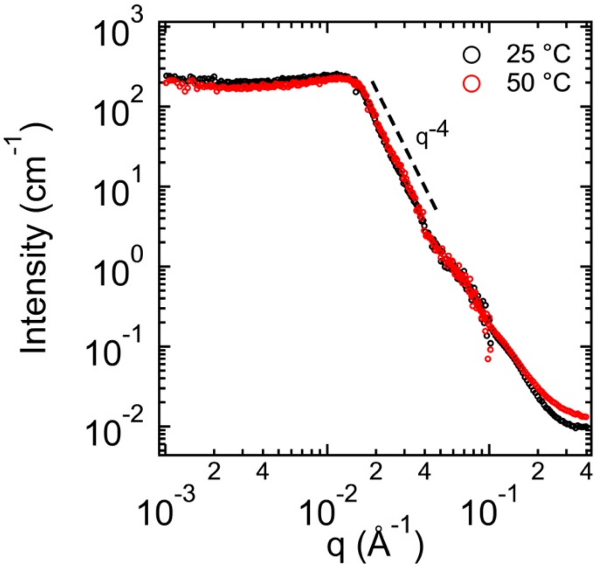

Supplementary Figure 5 Small angle X-ray scattering data (scattering intensity I(q) vs. scattering vector, q) obtained for thermogelling nanoemulsion at 25 °C (sol state) and 50 °C (gel state). The data represent desmeared scattering. The formulation contains 20% wt. isopropyl myristate (volume fraction is ~ 0.24) as an oil phase, 20% wt. emulsifiers (a mixture of Tween 80 and Span 80 with HLB = 13), 5% wt. PEG 400, Pluronic F127 (3.7 mM), and the remaining DI water. The hydrodynamic droplet diameter is 53 nm. Thermogelling behavior of a similar formulation is shown in Supplementary Figure 7 (NE with F127).

Thermal cycling. To investigate the thermal history effect of our thermogelling nanoemulsion, the rheological properties were monitored during two different cyclic tests. First, the formulation was subjected to a temperature ramp experiment from 10 °C to 60 °C (Supplementary Figure 6a) and then subsequently returning to 10 °C (Supplementary Figure 6b). The temperature ramp rate for both heating and cooling steps is 2 °C/min. In another set of measurements, the thermogelling nanoemulsion was subjected to multiple temperature jumps from 10 °C to 50 °C and from 50 °C to 10 °C (Supplementary

Figure 6c). The material was kept for 20 min at each step prior to the temperature jumps. The results indicated that the G' and G'' are not fully recoverable through the cooling step after the material turned into a gel in the heating step. In fact, after forming the gel at an elevated temperature, the structure still remains in the gel state (a weaker gel) when temperature returns to 10°C. A similar observation was previously reported for concentrated pure Pluronic aqueous solution.[8] The obtained results highlight the possible bridges formed among the hairy oil droplets and assembled Pluronic micelles at an elevated temperature (as depicted in Figure 1c).

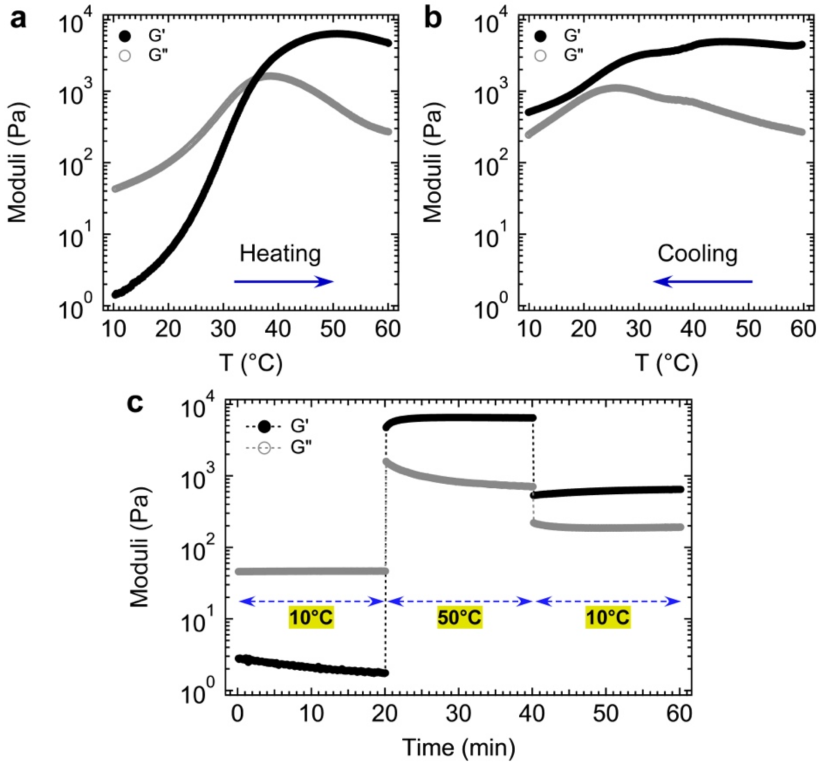

Supplementary Figure 6 Hysteresis during temperature cyclic experiments of the thermogelling nanoemulsion. Evolution of the dynamic moduli in a small amplitude oscillatory shear temperature ramp experiment during **a** heating from 10 °C to 60 °C and **b** cooling from 60 °C to 10 °C with a ramp rate of 2 °C/min. **c** Linear viscoelastic moduli against time of the measurement in a temperature jump from 10 °C to 50 °C and to 10 °C. The interval time is zero between the steps and measurements were performed at $\gamma_0 = 0.1\%$ and $\omega = 20$ rad/s. The formulation contains 20% isopropyl myristate as the oil phase, 20% surfactant as an emulsifier (a mixture of Tween 80 and Span 80 with HLB = 13), 5% PEG 400, 4.7% Pluronic (F127/F68: 6/1 g/g), and 50.3% DI water. All concentrations are in mass percentage. Hydrodynamic oil droplet diameter is ~ 53 nm with PDI ~ 0.12.

Thermogelling nanoemulsion using different Pluronic block copolymers. Thermogelling behavior of our canonical nanoemulsion suspension was investigated using different types of Pluronic (relatively similar length of polyethylene group), listed in table S1. Critical micelle temperatures (CMT) of the Pluronic aqueous solution were obtained from micro-DSC measurments at a fixed polymer concentration (4.6 mM, which is the local concentration of the copolymer used in the canonical system shown in Figure 2 in the main text). Supplementary Figure 7 displays the evolution of G' and G'' in a temperature ramp experiment for thermogelling nanoemulsions using different Pluronic as the gelator (F88, F127, F98, and F108, with a similar molarity of 3.7 mM (equivalent to ~ 4.6% wt. F127). Several observations can be

made from these results. First, thermogelling behavior of the nanoemulsion is consistent for other Pluronic copolymers with a similar PEO chain length and molecular weight. Second, nanoemulsions with F127 copolymer exhibited the highest viscoelastic moduli at room temperature which has the highest ratio of propylene oxide to polyethylene oxide repeat unit (PO/PE). To compensate for this behavior, we replaced some F127 with F68 in our canonical system (which was the main reason we selected the mixed gelators in the canonical formulation). In addition, the nanoemulsion with F88 has the highest gelation temperature (~ 45 °C), corresponding to its highest CMT value (32 °C).

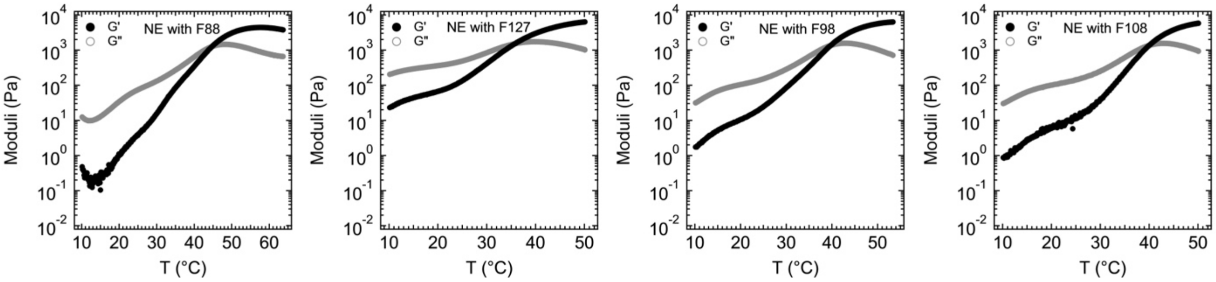

Supplementary Figure 7 Thermogelling behavior of the nanoemulsion with different copolymers (a similar molar concentration of the gelator). It contains 20% wt. isopropyl myristate as an oil phase, 20% wt. emulsifiers (a mixture of Tween 80 and Span 80 with HLB = 13), 5% wt. PEG 400, Pluronic (3.7 mM), and the remaining DI water. All nanoemulsions have a similar hydrodynamic droplet diameter of ~ 53 nm. The temperature ramps (2 °C/min) were conducted at small strain amplitude of 0.1% and frequency of 20 rad/s.

Supplementary Table 1. Characteristics of Pluronic block copolymers studied in this work.

Pluronic (gelator)	Mw (g/mol)	#PO[a]	#EO[a]	PO/EO	CMT, 4.6 mM (°C)[b]
F88	11,400	39	207	0.19	32
F127	12,600	65	200	0.33	20
F98	13,000	45	236	0.19	26
F108	14,600	50	265	0.19	26

[a] #PO and #PE are the number of repeat units for propylene oxide and ethylene oxide in the Pluronic chain.

[b] Critical micelle temperature (CMT) were recorded at the onset of endothermic peak in the micro-DSC results. CMT shown here are the copolymer solution with copolymer concentration of 4.6 mM (the local copolymer concentration in the thermogelling systems in Supplementary Figure 7).

Flow behavior of the thermogelling nanoemulsion as a function of temperature. To capture the viscosity growth as a function of temperature, a flow sweep test was applied to our canonical formulation. Supplementary Figure 8 displays the viscosity measurements under flow sweep test when temperature increases from 20 °C to 50 °C. We noticed that the viscosity monotonically grows as temperature increases until it reaches to nearly the same value obtained in Figure 6g at 50 °C.

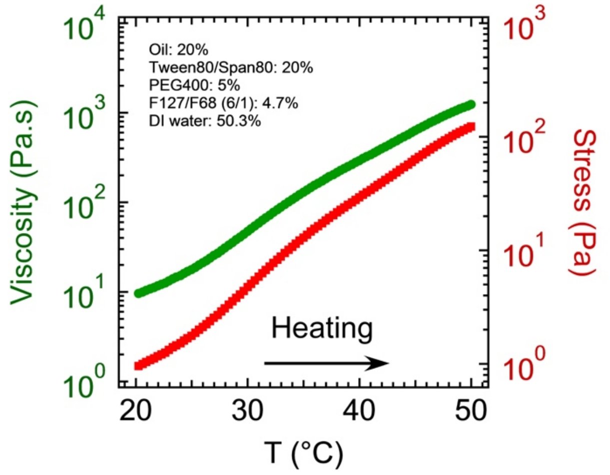

Supplementary Figure 8 Flow behavior of the thermogelling nanoemulsion as temperature increases. Oil droplet diameter is ~ 53 nm (a similar formulation presented in Figure 2 and Figure 6). Rheological measurements were conducted in a temperature ramp experiment (rate of 2 °C/min) and at shear rate of 0.1 s^{-1}. The nanoemulsion was loaded at 20 °C on the Peltier plate and a time sweep (strain = 0.1% and frequency = 20 rad/s) test was applied for 5 min prior to the application of the flow sweep test.

Dissolution experiments. The dissolution of ibuprofen (a model active pharmaceutical ingredient, API) from the nanoemulsion was studied at two different temperature, 10 °C and 37 °C (gelation behavior of the formulation without API is shown in Supplementary Figure 10c). The measurements were performed (Supplementary Figure 9) using the standard USP II (paddle) at 75 rpm. The UV measurements were obtained using Varian Cary 50 UV-Vis Spectrophotometer and *in situ* probe set. The nanoemulsions (~ 2 g) were manually added to the dissolution media prior to the measurements. DI water (~ 900 mL) was used as the dissolution media.

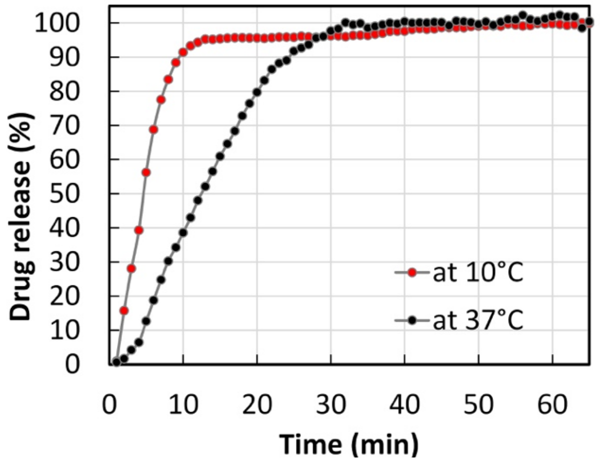

Supplementary Figure 9 Dissolution profiles of ibuprofen from the thermogelling nanoemulsion at two different temperature in DI water as a dissolution media. Gelation temperature of the nanoemulsion is ~ 22 °C. The composition is 25% wt. isopropyl myristate as an oil phase (contains 100 mg ibuprofen per 1 mL), 20% wt. emulsifiers (a mixture of Tween 80 and Span 80 with HLB = 13), 5% wt. PEG 400, 4.7% wt. Pluronic (F127/F68: 6/1 g/g), and remaining DI water. Samples were equilibrated prior to the measurements.

Effect of oil fraction and Pluronic concentration. Oil fraction and Pluronic concentration were systematically varied in the thermogelling nanoemulsions. Supplementary Figure 10 displays the evolution of G' and G'' and tan δ (tan $\delta = G'' / G'$, where tan δ is the phase angle) as a function of temperature for the nanoemulsion with different oil mass percentage of 15%, 20%, 25%, and 30% (equivalent to volume fractions of 0.18, 0.24, 0.30, and 0.35, respectively). The oil fraction can greatly change the gelation temperature (T_{gel}) and shear moduli. For example, the nanoemulsion with oil fraction of 30% wt. forms a gel at below the ambient temperature. Additionally, it has the highest G' values at 50 °C with the smallest tan δ value which is an indication of the stronger elastic properties. Similar behavior is observed with increasing Pluronic concentration into the nanoemulsion suspension. Supplementary Figure 11 displays the shear moduli as a function of temperature of thermogelling nanoemulsions with different Pluronic mass percentage from 3.9% to 4.7% to 5.4%, and to 6.2%.

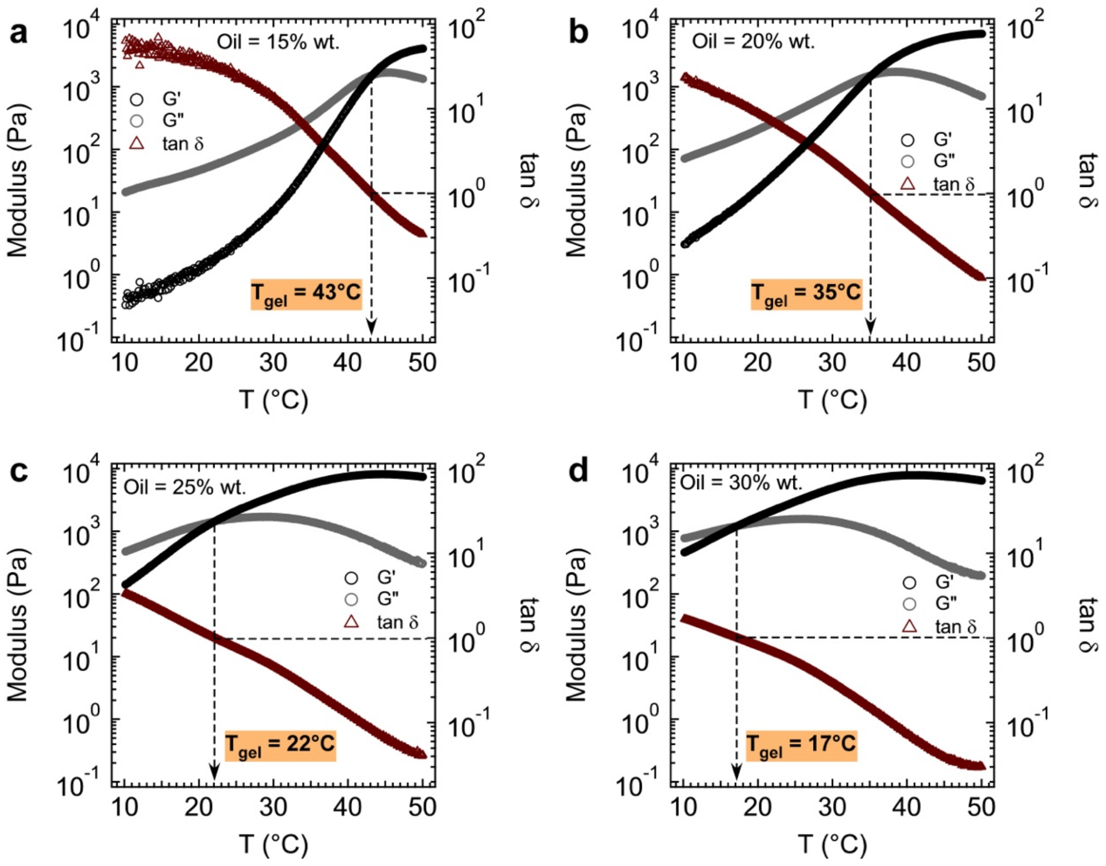

Supplementary Figure 10 Effect of oil fraction (isopropyl myristate) on the thermogelling behavior of the nanoemulsion while other composition kept the same; 20% wt. surfactant as an emulsifier (a mixture of Tween 80 and Span 80 with HLB = 13), 5% wt. PEG 400, 4.7% wt. Pluronic (F127/F68: 6/1 g/g), and the remaining is DI water. The hydrodynamic oil droplet diameters are **a**) 35 nm, **b**) 53 nm, **c**) 66 nm, and **d**) 89 nm. PDI is between 0.10 and 0.15.

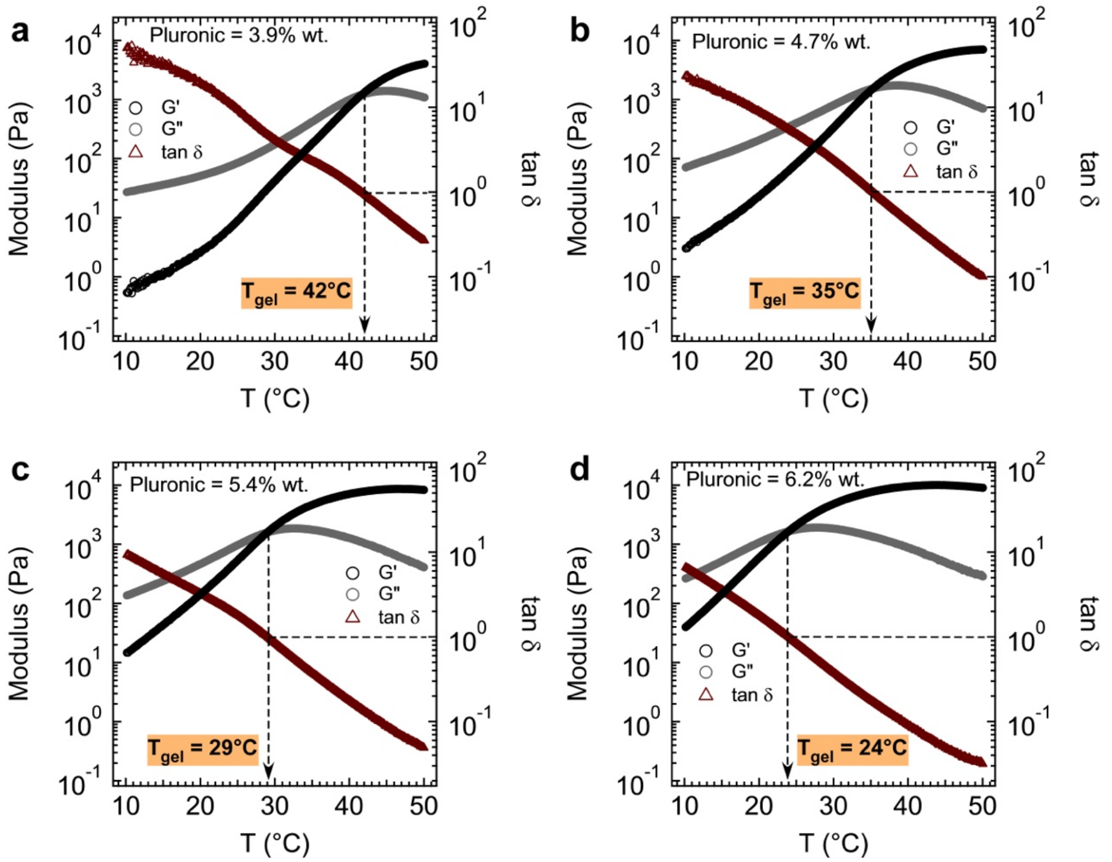

Supplementary Figure 11 Effect of Pluronic concentration (as the gelator molecule) on the thermogelling behavior of the nanoemulsion. The mass percentage of the composition is 20% oil (isopropyl myristate), 20% emulsifier (a mixture of Tween 80 and Span 80 with HLB = 13), 5% PEG 400, with Pluronic (F127/F68: 6/1 g/g) concentration of **a**) 3.9% **b**) 4.7% **c**) 5.4, and **d**) 6.2%, and the remaining is DI water. The oil droplet diameter is ~ 53 nm for all cases.

Supplementary references

1. Gupta, A., Badruddoza, A. Z. M. & Doyle, P. S. A General Route for Nanoemulsion Synthesis Using Low-Energy Methods at Constant Temperature. *Langmuir* **33**, 7118–7123 (2017).
2. Wooster, T. J., Golding, M. & Sanguansri, P. Impact of Oil Type on Nanoemulsion Formation and Ostwald Ripening Stability. *Langmuir* **24**, 12758–12765 (2008).
3. McClements, D. J. Nanoemulsions versus microemulsions: terminology, differences, and similarities. *Soft Matter* **8**, 1719–1729 (2012).

4. Morales, D., Gutiérrez, J. M., García-Celma, M. J. & Solans, Y. C. A Study of the Relation between Bicontinuous Microemulsions and Oil/Water Nano-emulsion Formation. *Langmuir* **19**, 7196–7200 (2003).
5. Cheng, L.-C., Godfrin, P. D., Swan, J. W. & Doyle, P. S. Thermal processing of thermogelling nanoemulsions as a route to tune material properties. *Soft Matter* (2018). doi:10.1039/C8SM00814K
6. Ilavsky, J. *et al.* Development of combined microstructure and structure characterization facility for in situ and operando studies at the Advanced Photon Source. *J. Appl. Crystallogr.* **51**, 867–882 (2018).
7. Helgeson, M. E., Moran, S. E., An, H. Z. & Doyle, P. S. Mesoporous organohydrogels from thermogelling photocrosslinkable nanoemulsions. *Nat. Mater.* **11**, 344–352 (2012).
8. Lau, B. K., Wang, Q., Sun, W. & Li, L. Micellization to gelation of a triblock copolymer in water: Thermoreversibility and scaling. *J. Polym. Sci. Part B Polym. Phys.* **42**, 2014–2025
