# Supplementary material for: Thermoresponsive nanoemulsion-based gel synthesized through a low-energy process
Source: Nat Commun. 2019 Jun 21;10:2749. doi: 10.1038/s41467-019-10749-1 (PMC6588569; doi:10.1038/s41467-019-10749-1)
Supplement: Supplementary file 3 — Description of Additional Supplementary Files [file 41467_2019_10749_MOESM3_ESM.pdf]

Description of Additional Supplementary Files

File name: Supplementary Movie 1

Description: Demonstration of sol-to-gel transition in the nanoemulsion formulation. Dripping of the room temperature nanoemulsion through a flat-tip 15-gauge needle into the water bath at 50°C. The composition of the formulation is 20% isopropyl myristate as the oil phase, 20% surfactant as an emulsifier (a mixture of Tween 80 and Span 80 with HLB = 13), 5% PEG 400, 4.7% Pluronic (F127/F68: 6/1 g/g), and 50.3% DI water. The hydrophobic dye (0.05 mg Nile red in 1mL oil) was incorporated in the oil droplet for the purpose of better contrast.
